# Gender disparities of chronic musculoskeletal disorder burden in the elderly Ghanaian population: study on global ageing and adult health (SAGE WAVE 1)

**DOI:** 10.1186/s12891-015-0666-3

**Published:** 2015-08-19

**Authors:** Emmanuel Kweku Nakua, Easmon Otupiri, Veronica Millicent Dzomeku, Ellis Owusu-Dabo, Peter Agyei-Baffour, Alfred Edwin Yawson, Gloria Folson, Sandra Hewlett

**Affiliations:** Department of Population, Family and Reproductive Health, School of Public Health, Kwame Nkrumah University Science and Technology, Kumasi, Ghana; Department of Nursing, Faculty of Allied Health Sciences, Kwame Nkrumah University of Science and Technology, Kumasi, Ghana; Department of Global Health, School of Public Health, Kwame Nkrumah University of Science and Technology, Kumasi, Ghana; Kumasi Collaborative Center for Tropical Research, Kumasi, Ghana; Department of Occupational and Environmental Health, School of Public Health, Kwame Nkrumah University Science and Technology, Kumasi, Ghana; Department of Community, College of Health Sciences, University of Ghana, School of Public Health, Korle-Bu, Accra Ghana; Noguchi Memorial Institute for Medical Research, College of Health Sciences, University of Ghana, Legon, Accra Ghana; College of Health Sciences, University of Ghana School of Medicine and Dentistry, Korle-Bu, Accra Ghana

## Abstract

**Background:**

Traditionally, non-communicable diseases including musculoskeletal disorders have not been a priority in low-and-middle income countries. The main aim of this paper is to assess age and gender specific burden by estimating the current prevalence of musculoskeletal disorders and associated risk factors in the elderly Ghanaian population.

**Methods:**

Between May 2007 and June 2008, the World Health Organization conducted a nationwide study on AGEing (SAGE) and Adult Health in Ghana. The study employed a multistage cluster sampling strategy to identify participants by stratifying the population by age and setting. A structured questionnaire was used for data collection. A Poisson regression model was fitted with robust error variance. Prevalence estimates took into account the complex survey design and sampling weights. Statistical significance was considered at p ≤ 0.05 significance level. Statistical analysis was performed with STATA version 11.2.

**Results:**

The prevalence rates of chronic back pain and chronic arthritis/joints pain were higher in women than men. The overall crude prevalence’s rates were 28.2 and 10.7 % for chronic back pain and chronic arthritis/joints pain respectively. Substantial differences existed between men and women in terms of socio-economic status, education level and occupational status. Women with primary education had a chronic back pain prevalence of 36.2 % (95 % CI; 29.2, 43.3) and chronic arthritis/joints pain prevalence of 15.8 % (95 % CI; 11.1, 20.6) while their male counterparts had prevalence rates of 29.0 % (95 % CI; 23.4, 34.5) and 9.8 % (95 % CI; 6.4, 13.2) respectively. Residence (rural and urban) did not appear to influence the prevalence of chronic back pain and arthritis/joints pain.

**Conclusion:**

Our findings suggest the existence of sex differences in chronic back pain and chronic arthritis/joint pain in the elderly population in Ghana after adjustment for demographic and socio-economic factors. It indicates the existence of inequalities in health between elderly men and women with women suffering more from chronic back pain and chronic arthritis/joints pain.

## Background

Due to the nagging effects of chronic diseases on the quality of life, chronic diseases remain a relevant public health problem and a medical challenge, with long term disability adjusted life years in both developed and developing countries. Per the United Nations (UN) estimates, the proportion of older people is expected to triple over the next 40 years, accounting for more than 20 % of the world’s population by the year 2050 [1]. The expected exponential increase in the elderly population would mainly be a product of rise in life expectancy, especially in developing countries. The associated rise is the incidence of non-communicable chronic conditions with consequent increase in morbidity and disability [2]. Chronic conditions impact heavily on economic productivity of families and have wider costs to health and social services [3].

Musculoskeletal disorders (MSD) are observed to be prevalent with pervasive impact especially in the elderly. They are the most common cause of severe long-term pain and physical disability, and affect hundreds of millions of people around the world [4]. MSD pain causes decreased quality of life among individuals, limits activity, and reduces functional capacity. To society, pain translates into considerable financial burden causing increased use of health services and medication, sickness absence and early retirement [5, 6]. Previous studies have emphasised the global magnitude of this problem. Mantyselka et al. (2001) concluded in their study that about 35 % of the Finnish population reported chronic pain; 40 % of all visits to general practitioners in this population is caused by pain [6]. A similar study in the United Kingdom showed that 79 % of those having chronic pain continued to suffer from the pain 4 years on. The annual recovery rate was only 5 % [7]. MSD, is ranked number one in chronic impairments in the United States and 1 out of every 4 people in developed and less developed countries reports chronic musculoskeletal pain [8]. There is however statistical dearth in terms of rates and other epidemiological measures of diseases in the developing world.

A number of population surveys have estimated the prevalence of individual musculoskeletal symptoms although accuracy in measurement remains a difficult challenge [9]. Fewer studies have however, considered the relative frequency of musculoskeletal disorders by gender. Those that have, suggest a considerable degree of overlap. The need for socio-economic factors to be considered also arises since they are known to play an important part in the occurrence of back pain, arthritis/joints pain and some general musculoskeletal disorders. The role of socio-economic factors in musculoskeletal disorders cannot be placed in the realm of oblivion and thus have to be explored fully. Traditionally, public health priorities have been assessed mainly by mortality statistics. In rapidly ageing populations, particularly those in developing countries, mortality statistics tend to be inaccurate or incomplete, and do not reflect those conditions that do not cause death but contribute chiefly to morbidity and disability [10].

The main aim of this paper is to estimate the current prevalence of musculoskeletal disorders in the elderly population in Ghana. The article also seeks to assess the level of socio-economic related inequality in the prevalence of MSD, and to suggest the extent and direction of equity in health care provision with respect to socioeconomic deprivation in providing evidence to guide the implementation of the National Ageing policy of 2010 to addressing this health burden in Ghana.

## Methods

### Sample

The data were obtained from the World Health Organization Study on Global AGEing (SAGE) and Adult Health survey conducted from May 2007 to June 2008. The aim of this multi-country study was to fill the data gaps for the older population in low-to-middle income countries.

SAGE is a longitudinal study with national representation and probabilistically selected. The study sampled persons aged 50+ years in Ghana, with comparison samples of young adult’s age 18–49 years in each country. The study employed a multistage cluster sampling strategy to identify participants and stratified by administrative region (Ashanti, Brong Ahafo, Central, Eastern, Greater Accra, Northern, Upper East, Upper West, Volta and Western) and setting (rural/urban), resulting in 20 strata. A total of 10–15 Enumeration Areas (EA) were selected from the strata according to size. Twenty households with persons aged 50 years and four households with person’s aged 18–49 years were then selected for interview. All persons aged 50-plus in ‘older’ households (households with at least one person age 50-plus years) were recruited to participate, whereas only one person was randomly selected in ‘younger’ households (households with no person aged 50-plus years) for the individual interview. If a selected individual was found to be incapable of completing an interview for reasons of health or cognition, a proxy questionnaire was completed. Standardized training in all aspects of the interview was provided to all interviewers. The survey was implemented as face-to-face interviews. Detailed sampling procedures have been explained in a methodology paper by Kowal et al., 2012 [11]. The questions related to MSD aimed at identifying socio-demographic and economic characteristics as well as symptoms of chronic diseases including back pain, arthritis/joints pain and general chronic pains were asked. Data were collected by means of a structured questionnaire. Ethical clearance was provided by the WHO as well as the Ethical and Protocol Review Committee of the University of Ghana Medical School. Informed consent was obtained from all participants.

#### Outcome measures

The outcome variables in this analysis were based on self-reported chronic health conditions by respondents. Back pain and arthritis/joint pain were the two outcomes of interest. Pains related to injuries from accidents such as falls, struck/hit by falling object and road traffic accidents by person were excluded. For each of these outcomes information of pain status was elicited either in two or more questions. Respondents were first asked whether they had been diagnosed with arthritis (a disease of the joints, or by other names rheumatism or osteoarthritis) and had sought medication for the last 12 months (yes/no) including the last 2 weeks. Respondents were then asked whether they had experienced back pain and the number of days it lasted during the last 30 days. Answers were dichotomized as the presence or absence of self-reported symptoms. To classify the back pain and arthritis/joints pain as chronic or other, a question on whether a respondent’s visit to health care provider was for a chronic (ongoing) condition, new condition, both or routine check-up was asked. Pain status was only classified as chronic pain if the visit was for an ongoing condition all new cases were excluded.

##### Covariates

Sex, age (50–59, 60–69, 70–79 and 80+ years) and marital status (never married, currently married/cohabitating, separated/divorced, widows) were some socio-demographic variables of interest. The questionnaire collected information on the work history of the respondents whether the respondent has ever worked for pay, type of work, place of work and for how long the respondent worked. The type of work of the respondents was classified using the International Standard Classification of Occupation (ISCO-88) [12]. The ISCO-88 has nine (9) categories (1. Legislators, senior officials and managers, 2. Professionals, 3. Technicians and associate professionals, 4. Clerks, 5. Service workers and shop and marker sales workers, 6. Skilled agricultural and fishery workers, 7. Craft and related workers, 8. Plant and machine operators and assemblers and 9. Elementary occupations) but for the purposes of this paper, the categories were re-grouped into four categories (Professional, Technical, Skilled agricultural and fishery workers, and Elementary occupation). Also considered were education (no formal education and some formal education), and wealth quintiles (poorest, poor, middle, and upper fifth and wealthiest) estimated from the household assets using the principal component analysis. The characteristics of men and women were compared by residence.

##### Statistical analysis

The percentage distribution of socio-demographic and socio-economic variables by sex and by setting (urban/ rural) was estimated as well as the prevalence of chronic back pain and arthritis/joint pains. Prevalence were estimated by setting as well as age-adjusted rates with 95 % confidence intervals (CI) estimated using the population structure of Ghana for the 2010 population census as the standard [13]. This analysis was restricted to respondents who had arthritis/joints pain or back pain, and who had sought treatment from the health facility for a chronic pain. A Poisson regression model was fitted with robust error variance. This regression technique allows for a conservative estimation of the relative risk when the outcome of interest occurs more than 10 % of the time, as in the case of back pain and arthritis/joints pain in this analysis [14]. Univariate analysis for each of the covariates was fitted. In the multivariate analysis, forward stepwise and backward stepwise models were performed. The result from the backward stepwise model was adopted. Models were fitted separately for each of the two outcome variables (chronic arthritis/joints pain and chronic back pain). Respondents who did not experience chronic back pain or chronic arthritis/joints pain were considered as the reference group for comparison with the disease outcome. Prevalence estimates took into account the complex survey design and sampling weights. Statistical significance was considered at p ≤ 0.05 significance level. STATA version eleven (11.2) (Stata Corp., College Station, Texas: StataCorp LP, USA) was used for all statistical analyses.

## Results

Table 1 presents the demographic and socio-economic characteristics of the study sample. From the sample, 1 925 were resident in rural areas while 2 799 were in urban areas. Similar mean age distribution was observed among rural men and women, while in the urban areas men were slightly younger. Majority of elderly men and women had no formal education; however it was observed to a greater extent in rural and urban women than men; 78.8 % versus 53.7 % (for rural women and men) and 60.4 % versus 36.0 % (for urban women and men).

**Table 1:** Demographic and socioeconomic characteristics by sex in urban and rural areas for the elderly population

	Rural(*N* = 2, 799)		Urban(1, 925)	
Socioeconomic variables	Women(1333) %	Men(1466) %	Women(1 044) %	Men(881) %
Mean age in years(95 % CI)	64.9(64.4, 65.5)	64.1(63.6, 64.7)	64.7(64.0, 65.4)	62.65(62.0, 63.3)
Age group (years)				
50–59	36.2	39.0	39.5	47.2
60–69	26.3	29.8	26.0	28.4
70–79	27.2	21.1	23.4	17.4
80+	10.3	10.2	11.2	7.0
Educational level				
No formal education	78.8	53.7	60.4	35.0
Primary Education	14.5	22.1	19.4	19.8
Secondary education	5.9	20.7	16.8	36.4
University/postgraduate	0.5	2.7	3.1	8.2
Missing	0.3	0.8	0.3	0.6
Marital Status				
Never Married	0.9	1.2	1.8	1.1
Currently married/Cohabiting	36.0	84.0	29.2	82.1
Separated/Divorced	16.4	7.4	20.5	9.3
Widowed	46.0	7.0	47.8	6.8
Missing	0.6	0.4	0.7	0.7
Type of Work^a^				
Professional	0.2	1.2	1.3	5.2
Skilled agricultural and fishery workers	67.4	82.5	42.4	61.0
Technical	4.6	5.1	10.3	13.5
Elementary occupation	10.2	2.2	29.1	10.1
Missing	17.7	9.1	16.9	10.2
Socio-economic status				
Poorest	27.5	27.0	11.8	6.0
Poor	27.2	22.9	14.0	10.8
Middle	23.3	22.4	17.1	15.0
Upper fifth	15.3	17.5	24.6	24.4
Wealthiest	6.6	10.2	32.2	43.6
Missing	0.1	0.0	0.4	0.2

^a^Professional includes-Legislators, senior officials, managers, clerks and professional; Elementary occupation includes Elementary occupation and Service workers and shop and market, Technical includes-Technicians and associate professionals, Craft and related trades workers, and Plant and machine operators and assemblers

With the marital status variable, currently married/cohabiting was prevalent for men living in both rural (84.0 %) and urban (82.1 %) areas while their women counterparts in both areas were more likely to be widowed. Skilled agricultural and fishery was predominant in both rural and urban settings for both men and women. In the urban areas, there were more women (23.6 %) working in the public sector compared with men (7.3 %). Majority of people in the rural areas were found in the poorest quintile and these proportions were similar for men (27.5 %) and women (27.0 %). The reverse was observed in the urban areas where there were a high proportion of men (43.6 %) in the wealthiest quintile than women (32.2 %).

**Fig. 1:**
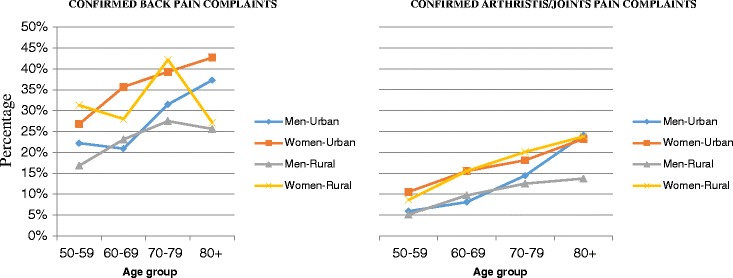
Crude prevalence of self-reported symptoms of chronic pain by age among men and women in urban and rural settings

Women in urban and rural areas reported a high prevalence of chronic arthritis/joints pain and chronic back pain (Fig. 1). There was an increasing slope of chronic arthritis/joints pain in both rural and urban women population as age increased; this was much steeper among women than men. A higher prevalence of chronic back pain was reported in urban areas by women. There was no real pattern for rural women with chronic back pain but it was more prevalent at 70–79 years. Generally, the prevalence of chronic arthritis/joints and back pain among rural men was low, even though one would expect a higher prevalence because of the farming activities in those areas.

### Aged-adjusted prevalence of musculoskeletal disorder (chronic back and chronic arithritis/joint pain) by sex

Table 2 presents age-adjusted prevalence of chronic musculoskeletal disorder among men and women with chronic back and chronic arthritis/joints pain. This is to project the prevalence and variance of prevalence between sexes. In general, chronic back and chronic arthritis/joints pain were more prevalent among women than their male counterparts.

**Table 2:** Age-Adjusted prevalence of chronic musculoskeletal disorder among men and women

	Men	Men	Women	Women	Men	Women
Variables	Total	Back pain	Back pain	Total	Joint pain	Joint pain
	N	% (95 % CI)	% (95 % CI)	N	% (95 % CI)	% (95 % CI)
Educational level						
No formal education	1095	21 (17.9, 24.9)	32.2 (28.4, 36.1)	1682	8.9 (6.3, 11.5)	14.1 (11.2, 17.1)
Primary education	498	29.0 (23.4, 34.6)	36.2 (29.2, 43.3)	396	9.8 (6.4, 13.2)	15.8 (11.1, 20.6)
Secondary education	624	23.3 (18.3, 28.4)	32.9 (25.1, 40.7)	254	9.3 (6.4, 12.2)	16.4 (10.3, 22.5)
Uni/postgraduate	112	24.5 (14.1, 35.0)	35.2 (16.6, 53.9)	39	9.9 (3.5, 16.3)	11.6 (0.6, 22.6)
Marital status						
Never married	27	20.0 (2.7, 37.4)	28.1 (9.4, 46.8)	31	10.3 (−0.9, 21.5)	9.4 (−2.9, 21.8)
Currently married/cohabiting	1954	24.3 (21.3, 27.3)	33.7 (28.1, 39.2)	785	10.0 (7.9, 12.1)	15.8 (10.6, 21.0)
Separated/divorced	191	23.8 (16.7, 30.9)	31.9 (26.0, 37.7)	433	7.4 (3.6, 11.2)	18.4 (14.2, 22.6)
Widowed	163	30.4 (19.1, 41.62)	32.9 (28.5, 37.2)	1113	14.1 (6.1, 22.1)	14.0 (10.9, 17.1)
Type of Work						
Professionals	64	28.4 (17.6, 39.2)	16.4 (3.9, 28.9)	15	5.7 (0.4, 11.0)	13.5 (−0.13, 27.2)
Skilled agricultural and fishery workers	1746	26.2 (23.1, 29.3)	32.9 (29.2, 36.6)	1341	10.6 (8.2, 13.0)	14.4 (11.6, 17.2)
Technical	193	17.9 (11.5, 29.3)	40.8 (31.2, 50.4)	168	7.8 (2.8, 12.7)	14.5 (6.9, 22.1)
Elementary occupation	121	17.8 (10.3, 25.2)	32.5 (27.3, 37.7)	441	14.7 (6.6, 22.8)	14.9 (10.7, 19.1)
Socio-economic status						
Poorest	449	12.9 (8.5, 17.4)	23.7 (18.0, 29.4)	489	6.5 (2.2, 10.9)	10.1 (26.9, 13.2)
Poor	430	23.2 (17.1, 29.3)	28.8 (23.2, 34.4)	509	9.4 (5.9, 12.9)	12.3 (9.2, 15.4)
Middle	460	37.0 (30.5, 43.5)	38.3 (32.4, 44.2)	489	9.7 (6.1, 13.3)	14.9 (10.7, 19.1)
Upper fifth	472	21.3 (16.8, 25.8)	38.3 (32.2, 44.4)	461	9.0 (6.3, 11.7)	17.6 (13.0, 22.1)
Wealthiest	534	24.9 (20.0, 29.9)	31.8 (25.8, 37.7)	424	14.1 (10.1, 18.1)	16.9 (12.4, 21.4)
Setting						
Rural	881	24.1 (20.4, 27.9)	32.2 (28.0, 36.4)	1044	10.2 (7.3, 13.1)	13.8 (10.7, 16.8)
Urban	1466	24.2 (20.4, 27.9)	33.0 (28.7, 37.3)	13 33	10.0 (7.4, 12.5)	15.6 (12.0, 19.1)

The prevalence of chronic back pain among women with primary education was slightly higher than those with university/postgraduate education 36.2 % (95 % CI; 29.2, 43.3) and 35.2 % (95 % CI; 16.6, 53.9) respectively. Also chronic arthritis/joints pain was slightly more prevalent with women who had secondary education than primary education 16.4 % (95 % CI; 10.3, 22.5) and 15.8 % (95 % CI; 11.1, 20.6). Even though men had lower prevalence compared with women, chronic back pain was more prevalence among men with primary education 29.0 % (95 % CI; 23.4, 34.6) while chronic arthritis/joints pain prevalent was evenly distributed across the education level (see Table 2).

There was no clear difference with the prevalence of chronic back pain and chronic arthritis/joints pain between rural (24.1 %) and urban (24.2 %). However, chronic back and chronic arthritis/joints pain were more prevalent among women in rural and urban areas when compared with men. Apart from the rural urban dichotomy, type of work was also a prominent determinant of the prevalence of musculoskeletal disorder. Professional men workers and women in technical skills had higher prevalence of back pain 28 % (95 % CI; 17.6, 39.2) and 40.8 % (95 % CI; 31.2, 36.6) respectively. The high prevalence of women in technical skills might be due to the combined role as a mother.

Men who were widowers had a higher prevalence of back and arthritis/joints pain while back pain was more prevalent among women currently married/cohabiting. However, those separated /divorced had a higher prevalence of arthritis/joints pains. Men and women in the middle wealth quintile experienced approximately similar prevalence of back pain. In contrast, women in the wealthiest category had a higher prevalence of arthritis/joint pain.

Table 3, shows the risk factors associated with chronic back pain and chronic arthritis/joints pain identified using Poisson regression models. The multivariate model indicated that women are more at risk of chronic back pain aIRR = 1.30 (95 % CI; 1.11, 1.53) and chronic arthritis/joints pain aIRR = 1.37 (95 % CI; 1.06, 1.78) compared with their male counterparts after accounting for the effect of other covariates. Men aged 70–79 and 80 years or above are aIRR = 1.41 (95 % CI; 1.18, 1.68) and aIRR = 1.40 (95 % CI; 1.11, 1.76) times more at risk of chronic back pain compare with the 50–59 years age group after accounting for marital status, occupation status and socio-economic status. An age gradient was found for arthritis/joints pain; the incidence rate ratio of chronic arthritis/joints pain increased with age for both men and women. However, there was no gradient for chronic back pain. There was a positive association between socio-economic status and the risk of chronic back pain and chronic arthritis/joints pain. The risk significantly increased with wealth quintile, those with chronic back pain in the middle quintile aIRR = 2.03 (95 % CI; 1.64, 2.50), the incidence rate ratio was about twice compared with the poorest quintile.

**Table 3:** Risk factors associated with chronic musculoskeletal disorder in elderly population, Ghana

	Chronic back pain				Chronic arthristis/joints pain			
	Univariate model		Multivariable model		Univariate model		Multivariable model	
Variables	IRR**	(95 % CI)	aIRR**	95 % CI	IRR	95 % CI	aIRR	95 % CI
Gender								
Men	1.00				1.00		1.00	
Women	1.36	1.20, 1.54*	1.30	1.11, 1.53	1.45	1.20, 1.74*	1.37	1.06, 1.78*
Age group								
50–59	1.00		1.00		1.00		1.00	
60–69	1.17	1.00, 1.38*	1.18	1.00, 1.39	1.48	1.15, 1.91*	1.50	1.15, 1.95*
70–79	1.43	1.21, 1.69*	1.41	1.18, 1.68	2.01	1.57, 2.57*	2.02	1.56, 2.62*
80+	1.36	1.09, 1.69	1.40	1.11, 1.76	2.30	1.69, 3.14*	2.39	1.74, 3.30*
Settings								
Rural	1.00				1.00			
Urban	1.01	0.86, 1.18			1.03	0.76, 1.40		
Educational level								
No formal education	1.00				1.00			
Primary education	1.03	0.88, 1.21			0.86	0.65, 1.15		
Secondary education	0.84	0.69, 1.02			0.79	0.61, 1.03		
Uni/postgraduate	0.87	0.59, 1.27			0.72	0.41, 1.24		
Marital status								
Never married	0.76	0.40, 1.45	0.76	0.38, 1.52	0.80	0.32, 2.04	0.73	0.27, 1.97
Currently married/cohabiting	1.00		1.00		1.00		1.00	
Separated/divorced	1.18	0.99, 1.41	1.10	0.92, 1.32	1.26	0.99, 1.61	1.15	0.88, 1.41
Widowed	1.34	1.17, 1.52*	1.10	0.93, 1.30	1.49	1.21, 1.83*	1.07	0.80, 1.43
Type of work								
Professional	0.95	0.55, 1.64	0.15	0.67, 1.96	0.56	0.27, 1.19	0.68	0.32, 1.45
Skilled agricultural	1.06	0.90, 1.24	1.15	0.97, 1.37	0.90	0.67, 1.22	1.04	0.76, 1.41
Plant	1.00	0.77, 1.29	1.08	0.83, 1.39	0.76	0.46, 1.25	0.84	0.50, 1.41
Elementary occupation	1.00		1.00		1.00		1.00	
Socio-economic status								
Poorest	1.00		1.00		1.00		1.00	
Poor	1.44	1.14, 1.83*	1.45	1.14, 1.84*	1.32	0.91, 1.91	1.32	0.92, 1.92
Middle	2.04	1.65, 2.52*	2.03	1.64, 2.51*	1.38	0.96, 1.99	1.38	0.95, 2.00
Upper fifth	1.58	1.23, 2.02*	1.62	1.27, 2.08*	1.43	0.99, 2.06	1.49	1.03, 2.17*
Wealthiest	1.46	1.15, 1.85*	1.61	1.26, 2.06*	1.68	1.15, 2.44*	1.95	1.33, 2.85*

Significance level * P ≤ 0.05 **IRR = Incidence Rate Ratio and aIRR = Adjusted Incidence Rate Ratio

## Discussion

The findings of this study suggest a high prevalence of chronic back and arthritis/joints pain (28.2 % and 10.7 % respectively), in a nationwide sample of urban and rural elderly men and women. The prevalence of chronic back and chronic arthritis/joints pain is greater among women than their male counterparts across the age groups. Among women however, the rates of chronic arthritis/joints and chronic back pains were observed not to show any variations across the age groupings. For the age group 60 years or above, the prevalence for arthritis/joint pain is relatively low for the urban dwelling women but high in the rural dwelling women, with equilibrium in rates, reached at 80+ years. Though a number of studies do not find same for study participants in their cases, others have reported a significant association of joint and muscle pain with gender. This was evidenced in a Cape Town study in which it was concluded that the higher prevalence of MSD in women, is a pattern recognized in many chronic musculoskeletal conditions including rheumatoid arthritis(RA), Osteoporosis(OA) and fibro- myalgia [15].

Chronic back pain and chronic arthritis/joints pain were observed to increase with age among women: while it was observed to be on a steep rise to 60 years in urban dwelling women, from where there is a small percentage rise in prevalence till equilibrium is reached at 80+ years; rural dwelling women exhibited an appreciable rise in prevalence with increasing age. This is consistent with the findings from a study by Urwin et al. (1998), they estimated the prevalence of musculoskeletal disorders at different anatomical sites. They reported in women, the prevalence of musculoskeletal pain at most sites increased with age up to 75 years and then reached a plateau [16]. Women at an older age are more exposed to various risks such as psychological stress, increase blood pressure and chronic diseases. Moreover, they are susceptible to earlier age strike by non-communicable diseases especially in less developed countries [17]. It is possible that most women in their older age continue to carry their grandchildren and participate in heavy work exposing them to chronic back pain and chronic arthritis/joints pain.

In men, it was observed that chronic arthritis/joints pain, though relatively lower in prevalence to that of women across same age group, showed general increasing prevalence with age. Across the age groupings for rural dwellers, the prevalence plateaus as against the steep rise in prevalence across the age groups for the urban dwelling males. Men in urban areas also reported a higher prevalence of chronic arthritis/joints pain, especially those 80 years or older. This observation is again in congruence with a study in Burkina Faso that showed a consistent relationship between age and the prevalence of NCD symptoms. However, the significance of this is paralleled by what appears, in terms of health conditions, to be evidence of early aging in the adult population. Chronic arthritis/joints pain and chronic back pain were unexpectedly high among the youngest, and their frequency increased steeply with age up to a prevalence of over 50 % starting in the group aged 55–64 years. These observations converge with those of other studies suggesting that NCDs are both more lethal and developing at earlier ages in sub-Saharan Africa [18]. Again consistency is elicited with a study in India, by, Hirve et al. (2010) they concluded that disability in all domains increased with increasing age [19].

In this study, a significant proportion of men (84.0 % rural and 82.1 % urban) are either currently married or cohabiting. This overwhelming proportion may be characterized by many factors such as biological, psychological and social factors [20]. Generally, men are encouraged to remarry after the loss of their spouse even at an older age while their female counterparts prefer either to care for their children/grand children or stay single. A study conducted in Uganda revealed that over half of the widowers remarry after the loss of their spouse [21]. Also socio-cultural factors limit the participation of men in child raising activities [22] in developing countries, hence most men remarry. The prevalence of chronic back and chronic arthritis/joints pain were higher among men who were widowers; 30.4 and 14.1 % respectively.

In contrast, a substantial proportion of women is widowed both in rural (46.0 %) and urban (47.8 %) areas than their males in rural (7.0 %) and (6.8 %) urban areas. It can be deduced from the demographic outcomes that women are living longer as there was a higher proportion of women aged 70 years or above than men (Table 1). This finding is consistent with the global change in the life expectancy of women [17, 23]. For many women, widowhood is a signal of unwanted and unplanned change in social and economic circumstances and in some cultures, it is associated with social stigma [23]. Older women living alone are more likely to be at risk of physical illnesses. The prevalence of chronic back pain of women who were widowed (32.9 %) was comparable to those currently married/cohabiting (33.7 %). On the other hand, women separated/divorced 18.4 % (95 % CI; 14.2, 22.6) differed prevalence of chronic arthritis/joint pain with those widowed 14.0 % (95 % CI; 10.9, 17.1). However, the longer life expectancy of women should not imply, adverse health outcomes [24].

Non-literacy was observed to be higher in rural dwelling women and men and this reflected in their socio-economic status. Thus the poor rural dwelling women's finding with regards to MSD emphasizes the reality of existing gender biases in relation to economic power, which may be among other factors, the product of lower levels of education and savings, and the poorer life-time earning histories of many women as indicated in the tables. Additionally, the care giving role of women in the society limits workforce participation, contributing to poorer health in older age [25–28]. Per this observation, the cyclical nature of poverty and ill health is highlighted. This study adds to the growing body of evidence that social deprivation is linked not only to mortality but also to morbidity. The results are consistent with those reported recently by the Tanzanian Ministry of Health and Social Welfare, which found that older people make up around one-third of all disabled people and people with decreasing levels of education [29]. Performance and functionality self-reports were similar across all SES quintiles. Self-reports on quality of life were not significantly influenced by socio-demographic variables.

### Strength and limitations

The exploration of the World Health Organization’s (WHO) Study on AGEing (SAGE) and Adult Health survey conducted from May 2007 to June 2008 data on Ghana, has allowed for the description of the sex disparities of chronic musculoskeletal disorder burden in an elderly Ghanaian population. The analysis was done with regards to socio-economic and demographic factors as well as symptoms of MSD. The aim of the disintegration analysis was to move beyond a basic comparison of sex differences in self-reported health, and instead begin to unravel the determinants of the differences and variations across contrasting settings and factors. The major strength of the study lies in its design and on the sample studied. Adult population health survey using specific and validated measures of function and generic measures of HRQOL is an adequate method to determine varied aspects of the burden of pain across diagnoses. This increases the generalizability potential of the results. A weakness of the study could be the concurrent diagnosis of other chronic diseases, which might not make our diagnosis, based on self-report accurate. The instrument used to assess self-reported health in this case a questionnaire, might not be able to fully capture people’s experiences and expectations for their health. However, this method for measuring health has been used as part of the World Health Survey in some 70 countries with robust results though. It makes both sound economic and social sense to keep the aged fit and healthy; therefore preventive interventions should be implemented in order to help reduce the costs of long-term care for chronic conditions [23].

## Conclusion

The estimated prevalence and risk factors in this paper highlights the urgency of adapting better health systems and services to meet the changing health-care needs of the aged in low-and middle-income countries, primarily by improving the capacity to prevent and manage back pain and arthritis/joints pains. Despite increased use of health services, there is evidence that health services do not necessarily meet all of the needs of the aged. They continue to experience substandard health treatments across a number of non-communicable diseases including back pain and arthritis/joints pain [30, 31].

In conclusion, there was a gender variation in musculoskeletal disorder that was significantly higher among women than their male counterparts. Age, marital status and socio-economic status were risk factors associated with musculoskeletal disorder. The risk of back pain and arthritis/joints pain were similar in rural and urban areas.

### Policy implication

Gender disparities in MSD provided by this analysis may be a useful advocacy material to garner increased effort by health and social organizations in the quest to implement gender-based policies in the National Ageing policy of 2010 in Ghana.

As a result of improved health status, life expectancy is anticipated to increase with time. This will lead to a higher proportion of elderly population than we currently have-demographic transition. Programmers and policies makers would have to develop and implement appropriate geriatric care policy and protocols to address this gap increases.

## Abbreviations

SAGE: Study on Global AGEing
MSD: Musculoskeletal disorder
WHO: World Health Organization
ISCO: International standard classification of occupation
RA: Rheumatoid
OA: Osteoporosis
NCD: Non communicable disease
HRQOL: Health related quality of life
